# Measuring FVIII Activity and Thrombin Generation Simultaneously With a Novel Point of Care Platform (EnzySystem HemA): Qualitative Usability Evaluation

**DOI:** 10.2196/77621

**Published:** 2025-10-16

**Authors:** Aernoud Bavinck, Saskia E M Schols, Wilfred Teunissen, Amy Shapiro, Nicole M A Blijlevens, Kyle Davis, Waander van Heerde

**Affiliations:** 1 Department of Hematology, Radboud University Medical Center, Nijmegen, The Netherlands; 2 Enzyre, Nijmegen, The Netherlands; 3 Hemophilia Treatment Centre Nijmegen-Eindhoven-Maastricht, Geert Grooteplein Zuid 10, Nijmegen, 6525 GA, The Netherlands, 31 24 361 1111; 4 Panton, Deventer, The Netherlands; 5 Department of Pediatric Hematology, Innovative Hematology, Indiana Hemophilia and Thrombosis Center, Indianapolis, IN, United States

**Keywords:** usability testing, point of care, thrombin generation, factor VIII activity, FVIII activity, user-centered design

## Abstract

**Background:**

Hemophilia A is an inherited bleeding disorder with an increased risk of excessive bleeding. People affected by hemophilia A with a severe bleeding phenotype are treated prophylactically with hemostatic agents. Monitoring treatment and adjusting it to the individual patient’s needs is complicated by the scarcity of laboratories equipped to perform relevant coagulation assays and the absence of point-of-care testing platforms. A novel near-patient testing platform called the EnzySystem HemA aims to address this issue by measuring factor VIII (FVIII) activity and thrombin generation within 1 hour of 100 µL of whole blood.

**Objective:**

This study assessed the usability of the EnzySystem HemA by health care professionals without prior training and gathered information on its perceived usability, effectiveness, usefulness, and acceptability.

**Methods:**

A qualitative, single-center formative usability assessment was performed. Participants performed an FVIII activity assay with a mockup of the EnzySystem HemA and were interviewed about its acceptability, usability, effectiveness, and usefulness. Video recordings of the sessions were obtained with permission and subsequently reviewed to assess usability; thematic analysis was performed on the interview transcripts.

**Results:**

A total of 7 health care providers participated, all unaffiliated with the EnzySystem’s developer. Participants included 1 (14%) pediatric hematologist, 3 (43%; pediatric) nurses, and 3 (43%; pediatric) nurse practitioners. Two (29%) of the participants also held management positions. Five (71%) participants successfully performed the FVIII activity assay as intended. Of the remaining 2 participants, one applied insufficient force when inserting the blood tube, whereas the other was unable to transfer the blood into the EnzySystem. This latter step caused difficulties in 3 (60%) other participants; however, they completed it correctly. Five (71%) participants did not dispose of the EnzySystem components as intended, primarily due to compatibility issues of the disposable parts with the inlet of the US standard medical waste containers. Five themes were generated from the interviews: *functional the way it is* focuses on the acceptance of the prototype despite suggestions for various improvements, *utility through faster results and increased access* and *potential for patient engagement* relate to the envisioned benefits of the platform, and *financial investment and return* and *ensuring accuracy* reflect on potential barriers for implementation.

**Conclusions:**

Despite significant usability issues, the need for faster and more accessible testing led participants to conclude that the current platform was acceptable for use, provided that proof of assay accuracy was demonstrated. The results of this study will form the basis for further development of the EnzySystem HemA. The effectiveness of any updates will be evaluated in future usability studies, which will also include people with hemophilia A as participants.

## Introduction

Hemophilia is a hereditary bleeding disorder, characterized by reduced activity of coagulation factor VIII (FVIII) [1-3]. To prevent and treat bleeding, treatment with FVIII concentrates or other prohemostatic agents is tailored to the patient’s bleeding phenotype and FVIII activity levels [4,5]. Solely relying on FVIII activity levels fails to accurately predict bleeding. The thrombin generation assay (TGA), which evaluates overall hemostatic capacity, may better predict bleeding risk [6-8] and may solve monitoring issues with novel treatments [9,10]. However, both FVIII activity and TGAs are limited to specialized coagulation laboratories.

Point-of-care testing (POCT), “testing at or near the site of patient care” [11], is increasingly used to enable accessible testing in various medical fields [12-16]. Despite advancements enabling miniaturized coagulation testing [17], POCT platforms for hemophilia A do not exist. The EnzySystem HemA, a novel POCT platform for FVIII activity levels and thrombin generation, may address this gap.

Historically, POCT implementation has been challenging because of limited alignment between developers and end users [18,19]. To address this issue, end users are involved early in the development process according to the principles of user-centered design [20], reducing the risk of late-stage user interface modifications. These modifications would necessitate substantial revalidation, particularly in advanced technologies such as microfluidic assays.

The development and technical validation of the EnzySystem HemA technology were recently published [21]. The version evaluated in this study is currently undergoing technical and usability optimization. This study aimed to identify which aspects of the platform’s user interface require modification to ensure safe and efficient use by health care providers (HCPs) without prior training. Specifically, it assessed usability, effectiveness, usefulness, acceptability, and use-related risks from the perspective of end users.

## Methods

### Study Design

This single-center, qualitative usability evaluation consisted of a usability scenario simulation and an in-depth interview. HCPs specialized in hemophilia care from the Indiana Hemophilia & Thrombosis Center (Indianapolis, IN) were invited via email. Nurse practitioners, (pediatric) hematologists, physician assistants, pharmacists, and nurses were considered for inclusion. Exclusion criteria included lack of hematological experience, inability to communicate in English, or unwillingness to provide informed consent. The study aimed to include approximately 6 participants with diverse professional backgrounds.

### Ethical Considerations

The study adhered to the Declaration of Helsinki. The study protocol was approved by the Ascension St Vincent Hospital Institutional Review Board (study ID RIN20240003) and registered on ClinicalTrials.gov (NCT06369740). All participants were provided written information outlining the study procedures, including video recordings, and provided written informed consent. Video recordings were accessible only to authorized personnel, including members of the study team, auditors, and quality assurance and quality control reviewers. The recordings will be securely stored for 15 years. Participants provided explicit consent for their research data—including video recordings—to be accessed by the study sponsor, who is also the developer of the EnzySystem. Participants were provided with US $50 compensation for their participation in this study when participation occurred outside their regular working hours.

The results are reported in accordance with the COREQ (Consolidated Criteria for Reporting Qualitative Studies; Checklist 1) [22].

### The EnzySystem HemA

**Figure 1.**
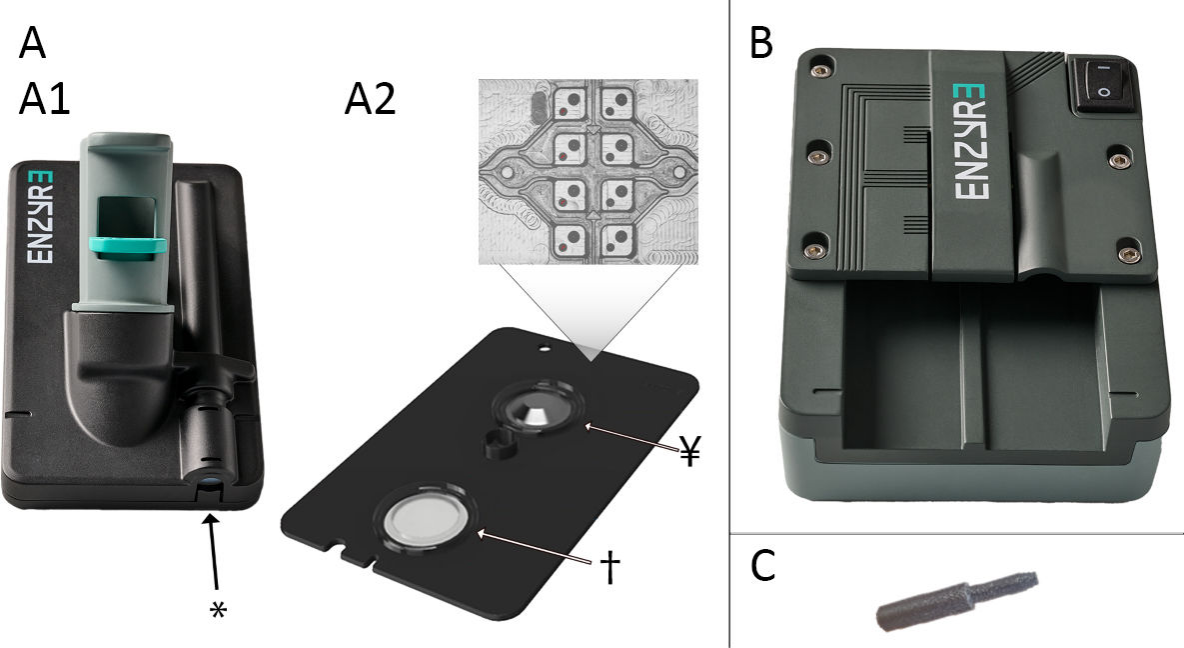
Overview of the EnzySystem components. (A) EnzyCard with (A1) the envelope with blood tube connector, incorporated syringe (*), and (A2) microfluidic cartridge with plasma separation membrane (†), blister filled with buffer (¥), and a magnified photograph of 8 of the detection chambers with spotted reagents. (B) EnzyPad processor. (C) Plastic plug used to depress the syringe in the EnzyCard envelope.

**Figure 2.**
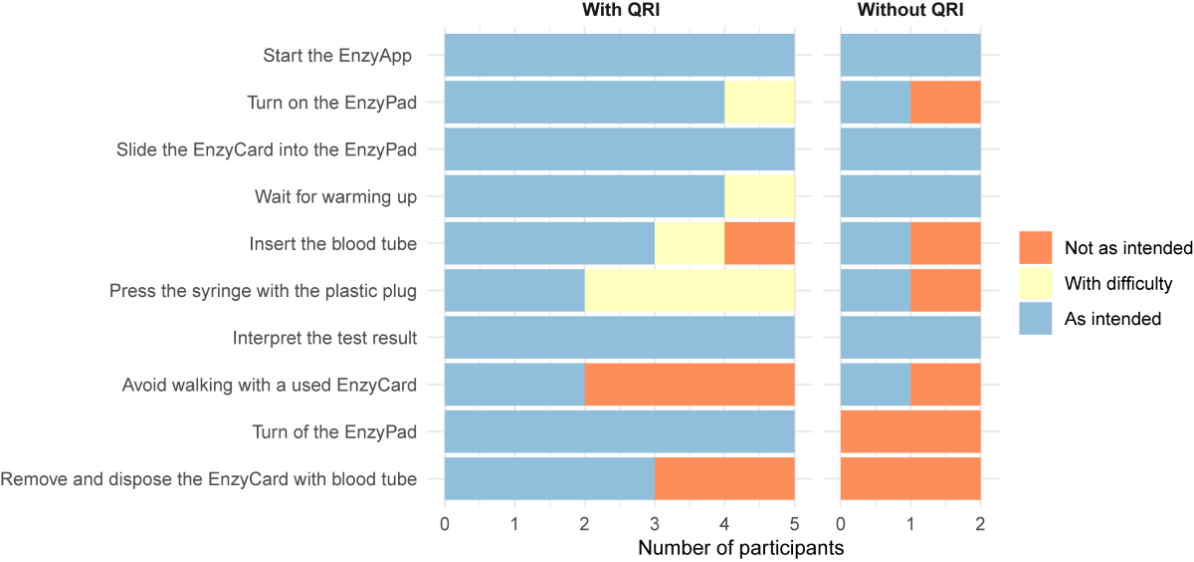
Correct task performance per user. QRI: quick reference instruction.

The EnzySystem is being developed by Enzyre B.V. as a novel platform for near-patient multiparameter testing. The EnzySystem HemA is designed to simultaneously measure FVIII activity levels and thrombin generation from 100 μL of whole blood within 1 hour. It consists of three integrated modules: (1) a disease-specific disposable EnzyCard (Figure 1A); (2) platform-specific software, the EnzyApp, that guides users through user tasks and displays results on a standard computer; and (3) the EnzyPad, a reusable processor into which the EnzyCard is inserted (Figure 1B).

The EnzyCard contains several elements: the external housing called the envelope (Figure 1A1) and an injection-molded microfluidic cartridge (Figure 1A2). Users only handle the envelope, which features a connector compatible with a standard 2.7 mL vacutainer for citrated blood. Upon insertion of a blood tube, blood is transferred into the EnzyCard by pressing a built-in syringe with an accompanying plastic plug. Inside the EnzyCard, plasma is separated from other blood components by a plasma separation membrane and enters the microfluidic cartridge. Capillary force drives plasma through the microfluidic channels, mixing it with buffer and dried-in reagents. Ultimately, the sample enters sixteen 110 nL detection chambers, where a chemiluminescent reaction occurs. Photons are generated proportionally to the analyte of interest and are quantified in real time by a sensor printed circuit board containing 16 single-photon avalanche diode optical sensors positioned above the detection chambers. The data are processed and sent via a USB cable to a computer for immediate display and interpretation.

In this study, a nonfunctional mockup of the EnzySystem HemA was used, consisting of the following components:

- A nonfunctional prototype of the EnzyPad (Figure 1B)
- An EnzyCard (Figure 1A) prepackaged in a sterilization bag (Elinic, Spain)
- A 3D-printed plastic plug (Figure 1C) packaged with the EnzyCard
- An EnzyApp graphical user interface represented by a PowerPoint presentation on a standard laptop, using a Wizard of Oz methodology [23] (Multimedia Appendix 1)
- A printed quick reference instructions (QRI) sheet (Multimedia Appendix 2)

A nonfunctional mockup was used to enable efficient usability evaluation while avoiding known nonusability errors associated with the current prototype. Early usability evaluation with nonfunctional mockups is a well-established approach, supported by the Food and Drug Administration’s human factors guidance [24]. Blood collection was excluded from the scenario, as this is a standard clinical procedure and the focus of the study was on platform-specific tasks.

Two included participants were deliberately tasked to perform the usability scenario without using the QRI to evaluate the intuitiveness of the EnzySystem HemA. They were purposefully selected based on their prior exposure to the platform in a focus group session on its potential use.

In addition to the EnzySystem HemA components, a standard 2.7-mL citrated vacutainer filled with water mixed with red dye, disposable nitrile hand gloves, and a standard medical waste container were used.

### Study Procedures

All included participants attended an individual usability testing session on August 22 or 23, 2024, in a meeting room at the Indiana Hemophilia & Thrombosis Center. Each session lasted 1 hour and was moderated by 1 member of the research team (WT), assisted by the first author (ABA).

Participants were seated at a conference table with the testing materials placed before them. Baseline characteristics were gathered with a structured interview. The moderator outlined the study goals and the components available for use during the scenario, following a predetermined script (Multimedia Appendix 3). Participants were then instructed to perform an FVIII activity assay and to clean up afterward. Participants assigned to not use the QRI were instructed to open the EnzyApp as a first step. Participants were encouraged to talk out loud [25] and were informed that the waiting times during preheating (normally 3 min) and running the assay (normally up to 1 hour) were shortened. The actual waiting times were disclosed at the respective steps.

Following the scenario, a semistructured interview using a preconstructed interview guide (Multimedia Appendix 3) was conducted to assess the perceived acceptability, usability, effectiveness, and usefulness of the EnzySystem HemA.

### Development of the Scenario and Interview Guide

The scenario and interview guides were constructed by the usability engineer in the research team (WT) based on medical input from the first author (AB). Both were refined based on 2 pilot sessions performed at the Radboudumc (Nijmegen, the Netherlands) with a nurse practitioner and a physician, both working in the field of hemophilia.

### Data Acquisition and Analysis

#### Usability Session

The usability sessions were recorded with 2 cameras. The video recordings along with field notes represented the main data sources. Task performance was documented for each participant in a Microsoft Excel framework. An experienced usability engineer (WT) assessed each task as either:

- As intended: Completed correctly without observable difficulty
- As intended with difficulty: Completed correctly without support; however, repeated attempts were required, the participant demonstrated clear uncertainty about how to perform the task or whether it was completed correctly, the task caused noticeable inconvenience, or, at the researchers’ discretion, completing the task required substantial effort
- Not as intended: Not completed, completed incorrectly, or required assistance

The overall assay was performed *as intended* if none of the tasks were assessed as *not as intended*. Tasks relating to the disposal of the EnzyCard were evaluated separately.

#### Semistructured Interview

The interview data were analyzed with Braun and Clarke’s [26] thematic analysis. The interviews were transcribed verbatim and coded inductively in Atlas TI version 23.4 by the first author (AB). Analytic field notes were recorded throughout the process. A coding book was kept during the data analysis (Multimedia Appendix 4). Interim findings and initial themes were discussed in research meetings with 3 of the investigators (AB, SEMS, and WVH). Codes concerning improvements for the EnzySystem HemA were counted to identify the most prevalent suggestions. Participants were not involved in checking the transcription and the final coding tree.

## Results

### Participants

A total of 110 HCPs were invited to participate. Twelve (11%) responded, of whom 5 (42%) were excluded: 1 was unable to attend physically, and 4 had professions that did not meet the inclusion criteria. Seven (58%) HCP participants were included in the study. Baseline characteristics are depicted in Table 1. None of the participants had used the platform before. However, 3 (43%) participated in a prior EnzySystem study involving focus group discussions on current coagulation testing and potential use cases.

**Table 1.** Baseline characteristics of participants.

	Age (years)	Gender	Profession	Participated in previous EnzySystem focus group discussions	Use of QRI^a^
HCP1^b^	39	Male	Pediatric nurse	No	Yes
HCP2	59	Female	Nurse practitioner, management role	No	Yes
HCP3	58	Female	Pediatric nurse practitioner	No	Yes
HCP4	38	Male	Nurse	Yes	Yes
HCP5	43	Female	Nurse practitioner	Yes	No
HCP6	34	Female	Pediatric hematologist	Yes	No
HCP7	61	Female	Nurse, management role	No	Yes

a QRI: quick reference instruction.

b HCP: health care provider.

### Usability Scenario

An overview of the success rate per task is depicted in Figure 2. Of the 7 participants, 5 (71%) performed the assay as intended, including HCP6 who did not use the QRI. The mean time to obtain a test result was 359 (SD 148) seconds, including two 20-second waiting periods for warming up and running the assay. Two (29%) HCPs were unable to perform the test as intended. One (14%) applied insufficient force when inserting the blood tube, expressing uncertainty on how much force could be applied before damaging the tube. The other, who performed the test without using the QRI, needed support to turn on the EnzyPad and insert the blood tube. This participant did not use the plastic plug to depress the syringe and transfer the blood sample into the microfluidic cartridge. Three (43%) other participants had difficulties completing this last task, hesitating over where to insert the plastic plug.

Only 2 of the 7 (29%) participants disposed of the EnzyCard as intended. Contrary to the instructions, 4 (57%) participants moved around with the used EnzyCard to discard it, and 4 (57%) removed the blood tube from the EnzyCard before disposal. The combined size of the EnzyCard and the blood tube constituted the main reason for difficulties with disposal, as together they did not fit into a standard US medical waste container.

### Interview on User Experiences, Perceived Acceptability, and Usefulness

Five interrelated themes were generated from the postscenario interviews: *functional the way it is*, *utility through faster results and increased access*, *potential for patient engagement*, *financial investment and return*, and *ensuring accuracy* (Table 2).

**Table 2.** Illustrative quotes for generated themes.

Generated themes	Illustrative quotes
Functional the way it is	
1.1	“I think a lot of the feedback is more to make it nicer, but I do think it’s functional the way it is.” (HCP6^a^)
1.2	“For clinic, I think it’s, would be fairly easy for a nurse to go through the whole steps, collecting the specimen, and prepping.” (HCP3)
1.3	“It’s smooth. The platform kind of lends itself to not running into user error, [...] it’s just a consistent chain. Even the program itself is pretty simple and straightforward. Everything goes together in one way.” (HCP1)
1.4	*“*This is, you know, this felt kind of how I expected it to. Feels like when you’re, when you’re collecting, it’s the same, same feeling popping the tube onto a needle, like when you’re collecting blood. So it’s a familiar feeling, to have that, and it’s a familiar shape too, it’s a vacutainer type of shape*.*” (HCP4)
1.5	*“*That [preference for automatic EMR interface] being said, exporting to a PDF would be fine. It would be a little bit more of a process, but exporting to a PDF and then printing that, and then you can scan it in into the printer work just the same.” (HCP1)
Utility through faster results and increased access	
2.1	“You don’t have to do any further processing with the blood tube at all, or sending it someplace, waiting on the courier to pick it up.” (HCP7)
2.2	“...after you do that [drop of the sample at the laboratory], you’re watching for results. You don’t know when they might populate, when they might not. So [with the EnzySystem] you would have the results readily available here to you, so you would not have to be sitting there watching every 10 to 15 minutes, clicking refresh to see if a result is coming through.” (HCP4)
2.3	“When this is sort of presented as, like, a point of care machine, 60 minutes is a very, very long time” (HCP1)
2.4	“I mean, you look at what you’re replacing. Like, if you told me you were running my blood sugar in 60 minutes, that would not be acceptable. But this is something that you’re replacing a test that’s trickier to get back on a timely manner.” (HCP2)
2.5	“Well, in the OR you need...would need like an instant result. Otherwise you have a patient that is bleeding.” (HCP5)
2.6	“And I can’t think of a scenario where, like, you know, a 60 minute will be like, [a] life or death decision.” (HCP6)
2.7	“If there is a bleed and I plan to give multiple factor infusions, it would be nice to know, like if the patient got replacement on one day...like got factor infusion on Monday and then the next day, you’re deciding whether to or not...whether or not to give a second dose, to give a minor or major dose, then it would be nice. We usually do that just going based on clinic and symptoms, but this would be helpful information.” (HCP6)
2.8	“Having this [EnzySystem] readily available for a pre-op patient would be great. Because I can check their pre level. I can run that while I am infusing. Collecting the post sample and by the time I’m done with everything this would be nearing completion on the first. And then I can run that. So by the time they are over at the hospital and I’m getting set up, everything’s good to go. We know what the result is.” (HCP1)
2.9	“Having this [the EnzySystem] available in the clinic, where we can get a result faster than what we can from the lab will help direct patient care faster, because sometimes they have to stay here until we get a result. If they can’t self infuse, then they have to wait for that result to decide or not...” (HCP5)
2.10	“And literally like to Kenya, we had all the people that we thought had hemophilia and we ran factor VIII tests first and then the people that didn’t were FVIII deficient, we ran factory IX tests. So it was a way, to kind of quickly do that. So I think if it was for...we were interested in a single test, [...] I don’t see really a downside to having it.” (HCP2)
Potential for patient engagement	
3.1	“My immediate thought with this is more the ability to use this at home. I think this would be a great tool for, for patients to have at home. So not all, but the majority of our patients, already have the ability to self infuse. So they stick themselves. They would be able to get a sample fairly easily. This is very user friendly. And again, with it being as small as it is, it’s not taking up a bunch of space. This would be great to have at the house if you are having breakthrough bleeding or something like that and if there is a concern that your prophylactic treatment is no longer working as it should, this would be a great way to provide some peace of mind and confirm whether or not levels are where they should be.” (HCP1)
3.2	“At home would be wonderful because that I, I know the, the families that I have with the CoaguChek monitors like it’s, it’s convenient because if they need to travel or anything like that, they can take the machine with them, and we get instant results, and I can tell them what to do with their medication instantly.” (HCP5)
3.3	“I think a child could [use the EnzySystem]...If you show how to do it. [...] So I think it helps them feel like they’re a part of their own care.” (HCP5)
3.4	“Some patients are not as reliable with regard to actually communicating information back, when they should. I guess you could consider only doing that [self testing] with patients that you would say, are more reliable, adherent to their treatment and that you know, are really going to be diligent about following whatever testing and ranges and things that you’ve set up.” (HCP7)
3.5	“If it’s [the blood tube] glass, breaking glass and causing potential injury to a layperson, and especially if you have kids are getting the test done, then proper disposal is going to be necessary.” (HCP3)
3.6	“It could be used outpatient home visits situation with our outreach nurses that actually go to the patient’s home and could do a test.” (HCP7)
3.7	“They would be in front of us and we would have that educational ability. And really, they would leave with a better understanding versus, you know, you get the results next day, then someone has to call them and talk to them. And so I think there’s a lot of information that the urgency, or that that immediate knowledge, when you’re in front of a provider would be, I mean, it would be very powerful.” (HCP2)
Financial investment and return	
4.1	“But even like blood sugar strips, you know, for diabetics are expensive. So I would anticipate this along with computer technology would not be inexpensive to use.” (HCP3)
4.2	“And that is the biggest struggle, is whether or not the insurance company will cover the device. I have some patients that still have to go to a lab because the insurance company, the insurance they carry won’t cover the device. So that’s the biggest burden.” (HCP5)
4.3	“Well, I think the hospital probably would not appreciate us bringing the device in to test our patients. Because that means they’re not getting a test, and they’re not getting the revenue for that test.” (HCP7)
4.4	“I think from the standpoint of resources, as far as thinking of factor as a resource, it’s very expensive. And if I can adjust it even a little bit and then get the same outcomes, I think that’s worth it.” (HCP2)
Ensuring accuracy	
5.1	“Yeah, I guess it [downsides of the EnzySystem] is just...it’s really determined because how accurate the point of care is.” (HCP3)
5.2	“I, I think, seeing the research study information, I think would be important for ensuring that the test information from the point of care and the conventional way would be equivalent in the result.” (HCP7)
5.3	“I would probably for the first time I may do this with a lab confirmed that, like they’re compatible, and then if that’s the case, then I will just use this device.” (HCP6)
5.4	“So if we have a known concentration, we run that through. All as well in the world and great.” (HCP1)
5.5	“Would there be something that you could put in here that would be like a little control, so I could see, like, their level, and then I’d see the control curve at the same time?” (HCP2)

a HCP: health care provider.

#### Theme 1: Functional the Way It Is

Although all participants suggested improvements (Table 3), the platform was considered usable in the current iteration (Quote 1.1, Table 2). The primary areas for improvement involved disposability, revising specific terminology in the QRI and graphical user interface, automating blood transfer into the EnzyCard, introducing feedback to verify task execution, and enabling automatic results export to the electronic medical record (EMR). Beyond these suggested improvements, the handling was considered sufficiently straightforward to be used in a clinic by HCPs or by patients (Quote 1.2, Table 2).

Participants attributed the experienced simplicity to the components fitting together in one way, making it intuitive and reducing error risk (Quote 1.3, Table 2). In addition, inserting the blood tube was perceived as analogous to using a regular connector during venipuncture (Quote 1.4, Table 2), which enhanced some participants’ confidence in performing the task correctly.

In agreement with the participants’ overall acceptance, most suggested improvements were framed as optimization rather than obligatory updates. The comments on potential connectivity were exemplary. All participants strongly preferred automatic EMR integration, but most would accept manual data import (Quote 1.5, Table 2). Notably, only 1 HCP deemed automatic export mandatory, as manual export would increase the risk of user error.

The participants highlighted the EnzySystem’s advantages over current testing as a reason for its acceptance.

**Table 3.** Number of participants suggesting specific improvements and requirements for the EnzySystem HemA.

Area for improvement	Count, n	Illustrative quote (participant)
Improve ease of handling		
Improve disposability	5	*“*If this is not reusable, it just seems like it’s going to be cumbersome for a lot of this disposal. So I don’t know what is in there, but, I mean, it’s big, if we do every test and we’re throwing this away, that’s a lot of pieces to be thrown out.” (HCP3^a^)
Streamline pressing the syringe^b^	4	“This [Plastic plug] didn’t feel like it fit very well. So it seemed clunky. But other than that, no. It would be nice to have it kind of already in there, and then you just engage it because, like, what if I dropped this or lost it or whatever?” (HCP2)
Refine blood tube handling	2	“Taking out [was difficult] ... More out than in. But a lot of it... I wasn’t sure how much to keep pushing.” (HCP6)
Incorporate automatic continuation of the GUI^c^	2	“Like I have used point-of-care testing, [...] so normally, once you put the cartridge in, it automatically runs itself. So to know the click ready on the screen, I think I wouldn’t want to do that...” (HCP5)
Improve cleanability	1	“Stainless or, you know, or something that that doesn’t have a lot of, like, little cracks and that kind of thing. These little lines, I know are... make it look nice, but, you have to clean it.” (HCP2)
Make sliding in the EnzyCard smoother	1	“I wish that the joining of the two, it felt just a little bit smoother.” (HCP1)
Prevent use errors		
Enhance understandability and consistency of instructions	7	“It was a little bit weird understanding that this is... this was a EasyPad [sic]. [...] It doesn’t say easy pad on it. And calling this an EasyCard seems a strange name to me because, it is not really cardlike.” (HCP7)
Add feedback mechanisms	5	“Just a little positive feedback once you finished that [pressing the syringe], such as a click, once you finish pressing in the syringe.” (HCP4)
Add handling instructions	4	“It felt firm going in [with plastic plug], so it’d be nice that... to make note that you will feel that firmness in there but continue on. Because I felt hesitant...” (HCP3)
Automate the blood volume transferred into the EnzyCard	2	“And also control that like, like blood that goes into the thing. [...] Because if I didn’t push that all the way in, would that still be 100 microliters.” (HCP6)
Label components	2	“What would be nice, because I know it’s prototype, but add more color to that to be easy to identify where the pieces go.” (HCP3)
Add a trigger release for sliding out the EnzyCard	1	“Given that you are handling a bodily fluid and maybe nice if it’s more of a trigger release. So if you had that locking mechanism where it latched and something that you can press release that, so that you’re not applying some arbitrary, force.” (HCP1*)*
Invert the direction of the blood tube connector	1	“So you insert the card this way, right. You’re applying an opposing force with the tube in the opposite direction. If it were possible, then might be nice if this were switched to the other direction.” (HCP1)
Prevent blood tube removal	1	“...if you wanted to remove the element of potential splash, you could put something inside of this tube during the manufacturing process, so it’s a one way.” (HCP1)
Protect from damage		
Enhance resistance to damage	2	“...the risk for it being dropped and broken and then repair. So for you making sure it will sustain falling and children.” (HCP3)
Prevent losing the plastic plug	2	“You could open this package up and potentially lose that little white thing and then you don’t have it.” (HCP7)
Enhance results interpretation		
Show past results	3	“What would be nice if this is this has been going on with the patient to see a trend and the numbers of past tests that we have.” (HCP3)
Add axis information	2	“If the scales are added, that may be a little more benefit to a physician, than a nurse, where I’m just kind of looking at the numbers.” (HCP4)
Add reference values	2	“I guess, if it was within range or. [.] Like, if it’s within range, like, do we need to do anything if it’s not within range, then an alert, a red alert.’ (HCP5)
Add a countdown clock	1	“A countdown clock would be great.” (HCP2)
Blood tube compatibility		
Prevent wasting unused sample volume	4	“In particular with babies, it would... might be hard to get that amount of blood that’s required, for the 2.7 ml tubes.” (HCP5)
Facilitate using other citrate concentrations	1	“If, for whatever reason, say, normal tubes were on backorder, we found a supplier that had a tube with a different concentration […]. Would the machine tolerate that?” (HCP1)
Data management and connectivity		
Enable automatic export to EMR^d^^,^^e^	6	“I mean it would need to interface into something, because I would hate to superimpose the numbers. And 45 is different than 54.” (HCP2)
Add a wireless connection	3	“So, I see, I see that it’s plugged in. It’s obviously a wired device. I don’t know if wireless is an option.” (HCP2)
Couple results to the patient ID	3	“But no, actually, I would want this to be connected to a patient ID because, even if this is not going to connect to the EMR, at least if there is memory of, like this [is the] patient and these are the results*.*” (HCP6)
Combine the results with treatment data	1	“...it would be nice if [...] patients could put in their last infusion and the amount [...], like WAPPS Hemo, the PK... […] If you could tie this to that somehow.” (HCP2)
Other		
Add additional assays	3	“Probably would like factor nine [FIX] and VWD.” (HCP6)

a HCP: health care provider.

b Also mentioned in preventing use errors; however, quotes in this category indicate that the usability could be more convenient rather than less prone to error.

c GUI: graphical user interface.

d EMR: electronic medical record.

e Some comments relate to preventing use errors; all comments about the automatic export of data were categorized under this code.

#### Theme 2: Utility Through Faster Results and Increased Access

Throughout the interviews, the primary reasons for the perceived utility of the EnzySystem were its potential to deliver faster and more accessible test results. The compatibility of the platform with whole blood combined with its near-patient design would enable streamlined and rapid testing (Quote 2.1, Table 2) and more predictable result availability (Quote 2.2, Table 2).

Interestingly, participants expected near-patient tests to be faster than the EnzySystem HemA (Quote 2.3, Table 2). However, their experiences with FVIII testing and understanding of its complexities increased acceptance of the required testing time (Quote 2.4, Table 2). Nonetheless, some participants considered shortening the testing time critical for use in a surgical setting and at the emergency department to guide treatment decisions (Quote 2.5, Table 2). However, others noted that the turnaround time would not limit clinical use in these situations because patients typically remain in a care facility for over an hour when receiving care (Quote 2.6, Table 2).

The most significant potential for the platform was envisioned in 3 types of situations. First, enhanced accessibility would enable personalization of treatment by performing testing in scenarios where it is currently not available (Quote 2.7, Table 2). This would be useful during at-home treatment for a bleed, before high-risk activities, to tailor prophylaxis, and after surgery. Second, the platform could streamline standard monitoring situations, such as presurgical assessments (Quote 2.8, Table 2) and regular clinic visits (Quote 2.9, Table 2). Finally, increased access could facilitate the diagnostic process in patients with suspected bleeding disorders, especially in low-resource countries (Quote 2.10, Table 2). However, some HCPs contended that the EnzySystem HemA might be unsuitable for diagnostic purposes, as additional laboratory tests beyond FVIII and TGA are often required. Consequently, blood samples would be sent to a laboratory regardless of using the EnzySystem.

#### Theme 3: Potential for Patient Engagement

Most participants anticipated opportunities for patient engagement through the EnzySystem HemA. First, home-based self-testing would allow for testing as needed and provide peace of mind for patients (Quote 3.1, Table 2) while eliminating the need for travel (Quote 3.2, Table 2). In addition, involving patients in testing was suggested to enhance their overall engagement with treatment (Quote 3.3, Table 2). However, self-testing might not be valid for all patients due to differences in patients’ technical skills, availability of equipment, insurance coverage, and reliability (Quote 3.4, Table 2). The significance of these factors depends on the features of the final product. For example, automatic export to the EMR could reduce the criticality of patient reliability. While the EnzySystem HemA was considered easy to use, the need for clear instructions and operational safety was further emphasized for patient self-testing (Quote 3.5, Table 2).

Even if home testing would not be feasible for all patients, increased patient engagement could be achieved by equipping outreach nurses with the EnzySystem HemA (Quote 3.6, Table 2) and by reducing the time to result in the clinic (Quote 3.7, Table 2). Observing the test result emerge on a screen during appointments could enhance understanding of the result, particularly when reference ranges are indicated. In addition, having results available for discussion during appointments was considered advantageous compared with calling patients after they leave the clinic.

#### Theme 4: Financial Investment and Return

One factor that could influence the use of the EnzySystem HemA was the cost and insurance coverage of the platform. Based on experience with other POCT platforms and the utilization of computer technology, 1 participant expected the costs of testing to be high because of typically expensive disposables (Quote 4.1, Table 2). Two other participants also indicated that costs would influence the device’s acceptability.

Arrangements regarding testing costs would impact implementation. Insurance companies have been reluctant to cover POCT costs, necessitating some patients to continue visiting laboratories for assays that could be performed using POCT platforms (Quote 4.2, Table 2). Furthermore, hospitals might prefer using their own conventional tests or acquiring an EnzySystem HemA themselves for in-house use, instead of using patient- or HTC-owned devices. This approach enables them to maintain testing revenues (Quote 4.3, Table 2).

While some participants raised questions about the platform and maintenance costs, others thought that increased access to testing would ultimately reduce overall health-related costs. Given the high costs of factor products used to treat hemophilia patients, reducing factor usage without increasing the risk of bleeding is highly desired. The 2 participants in management roles believed that adjusting treatment based on more frequent testing could prevent unnecessary administration of factor products and associated costs (Quote 4.4, Table 2).

#### Theme 5: Ensuring Accuracy

Across all interviews, the importance of ensuring accurate results was emphasized (Quote 5.1, Table 2). Participants indicated that they desired proof of accuracy before using the EnzySystem HemA (Quote 5.2, Table 2), as incorrect assay results could misdirect treatment or cause unnecessary concerns, such as fear of inhibitor development. Participants would study the results of clinical validation trials, which could offer sufficient proof of accuracy. However, some providers would still compare the EnzySystem Hem A’s results with those obtained with conventional coagulation tests as independent accuracy confirmation (Quote 5.3, Table 2).

Providing control samples was suggested as an additional method for accuracy validation (Quote 5.4, Table 2). Several participants indicated that access to samples with known FVIII activity, which could be sent with a batch of EnzyCards, would be sufficient for periodic quality control. Similar quality control strategies are used for other currently available point-of-care devices. One HCP noted that ideally, an internal control would be performed automatically with each run to provide assurance of the run’s validity (Quote 5.5, Table 2). This last suggestion would also provide confirmation of having correctly executed the assay. Several other participants suggested feedback mechanisms to ensure correct assay performance (Table 3). This consistent emphasis on validation highlights the necessity of ensuring result accuracy before implementing the EnzySystem HemA.

## Discussion

### General Results

In this first usability study of the EnzySystem HemA with HCPs, the overall usability and perceived utility of the platform were high. While the platform was considered usable, 2 participants were unable to use the EnzySystem as intended due to critical issues with the user interface. In addition, there is a uniform need to improve the disposability of the EnzyCard, as most participants did not dispose of it as intended. The platform’s perceived benefits over existing systems mitigate the concerns about its shortcomings. However, evidence demonstrating the platform’s reliability and accuracy is needed before implementation.

### Comparison With Previous Findings

Studies assessing human factor aspects of point-of-care in vitro diagnostics are scarce [27]. To our knowledge, no previous studies have evaluated the usability of products comparable to the EnzySystem HemA. However, research on attitudes toward POCT in general provides relevant insights. Notably, concerns about accuracy are not unique to the EnzySystem HemA. In a systematic review of primary care providers’ attitudes, uncertainty about accuracy was a key limitation of POCT [28]. Checking POCT results with conventional methods, as was suggested in this study, is also applicable to POCT in general [29]. Quinn et al [30] found that concerns about accuracy, data management, costs, and quality assurance were key barriers to POCT adoption in hospital settings, consistent with the findings of this study. However, regarding the EnzySystem HemA, 2 of the 7 (29%) participants suggested that implementation might reduce factor product use and related costs, aligning with broader discussions on the benefits of personalized medicine in hemophilia A [31-33].

The main envisioned benefits of the EnzySystem HemA also align with those described for POCT in general: shorter time to initiate treatment [29,34-37], improved communication with patients and patient engagement [29,34-37], and improved access to testing [35-37]. However, as no POCT is available for hemophilia A care at this time, the potential implications of these advantages, especially in this field, were described for the first time in this study.

Compared to most POCT platforms, a limitation of the current EnzySystem HemA is the need for a venipuncture, which limits the potential applicability for patient self-testing, as suggested in this study. An EnzySystem version compatible with a capillary blood collection system is planned for future development, but its usability for patients will require dedicated evaluation.

### Methodological Reflection

An experiential, inductive thematic analysis informed by a constructionist epistemology was used. Rather than seeking reproducible or externally generalizable results, the aim of qualitative research is to gain an in-depth, contextual understanding of participants’ experiences and the factors underlying the perceived acceptability and usability of the EnzySystem. This approach recognizes that both the participants’ perspectives and the researchers’ interpretations are influenced by subjective perspectives and that meaning is co-constructed throughout the research process.

To enhance the credibility of the analysis, quotes from participants were added to support the claims made. A codebook was maintained and is provided as supplementary material to communicate interpretative decisions and enhance methodological transparency (Multimedia Appendix 4). In addition, codes related to improvements of the EnzySystem Hem A were presented along with their frequency of mention (Table 3). While such counting techniques are uncommon in reflective thematic analysis—as frequency does not necessarily reflect the perceived importance of an issue [38]—they were included to highlight which improvements were most widely desired. Ultimately, however, the credibility of our findings rests on their ability to convincingly represent the participants’ experiences with the EnzySystem HemA and its perceived utility.

An inductive coding strategy was chosen, as this was the first usability evaluation of the platform, and no comparable platforms are available. However, the themes generated in this study relate to those commonly found in usability frameworks. For example, facets of Morville’s Usability Honeycomb [39], such as usability, usefulness, and credibility, relate strongly to themes 1, 2, and 5, respectively. As such, the generated themes do not exist in a theoretical vacuum but relate to the existing theoretical frameworks.

### Reflexivity

In reflective thematic analysis, it is acknowledged that researchers inevitably bring their own norms, values, and interpretive frameworks into the processes of data collection, analysis, and reporting [40]. Reflexivity in this study focused on the first author (AB)—who performed the coding and theme generation and assisted during the use scenarios—and the human factor engineer who moderated the scenarios (WT).

AB is a White Dutch male, a medical resident in internal medicine, and a PhD candidate at the Radboud University Medical Center (Radboudumc) in Nijmegen. He is employed by the developer of the EnzySystem during his PhD trajectory but holds no financial interests in the platform’s development. He received formal training in qualitative research, and his work focuses on personalized care in hemophilia A. He has no formal background in engineering or technological development.

WT is a White Dutch male senior product designer employed by Panton (Deventer, the Netherlands), a company specializing in medical innovation design. He has extensive experience in medical innovations, although this was his first project in the field of hemophilia A.

Both AB and WT had no prior relationship with any of the participants.

### Study Limitations

#### Study Setup and Contextual Considerations

Several factors limit the transferability of this study’s findings to real-world scenarios. First, the study was conducted in an office setting, which may differ from real-world clinical conditions. During use in practice, users must perform the assays while managing concurrent tasks, cope with environmental distractions typical of clinical settings (eg, maintaining communication with patients), and work under time pressure inherent to outpatient care. These factors may negatively impact user performance compared to the findings in this study. In addition to office use, several participants suggested that the EnzySystem HemA might be used in a laboratory environment, where the impact of unexamined environmental influences could be more pronounced.

In addition, a nonfunctional prototype of the EnzySystem HemA was used, and no real blood samples were collected. This approach was chosen to avoid invasive blood sampling and focus on usability aspects rather than technical validation, especially because the current prototype still has some known technical flaws. However, venipuncture is a critical step for performing the assay, and integrating it into the use scenario could uncover additional and significant usability issues.

Furthermore, as participants engaged in the use scenario only once, no information was obtained regarding learnability and memorability. As implementation of the EnzySystem HemA will involve repeated use by the same HCPs, these aspects should be explicitly addressed in future evaluations to provide a more comprehensive assessment of the platform’s long-term usability.

Finally, the findings of this study are specific to the contextual factors of the Hemophilia Treatment Center involved. While generalization is not the primary aim of qualitative research, it is important to consider that results may differ in other settings due to variations in treatment access, testing availability, and the degree of care specialization.

#### Potential Sources of Bias

The convenience inclusion procedure in this study may have favored the inclusion of HCPs with comparatively greater openness to research participation and new technologies. In addition, several factors may have biased the perspectives of the participating HCPs. For practical reasons, the assay’s waiting time was shortened. Although participants were informed of the actual waiting time, their experiences may have differed had the real waiting time been implemented.

Furthermore, participants were informed that this study was conducted by Enzyre, the company developing the EnzySystem HemA, and that a company-affiliated assistant was present during the study. While participants primarily interacted with the lead moderator and the assistant was formally trained in qualitative research—including methods to avoid suggestive or leading questions—some participants may still have adjusted their responses to be more socially desirable.

Regarding the interpretation of the results, it should be acknowledged that in qualitative studies, the influence of researchers on participants and interpretation cannot and should not be eliminated [40]. However, to mitigate the risk of conflation of company interests with the analysis, the primary analysis of the video recordings to assess task performance was performed by the unaffiliated scenario moderator. Furthermore, the full investigative team, including 5 members not affiliated with the developer, critically reviewed and approved the final analysis in the manuscript. Notably, the unaffiliated moderator had independently analyzed all use scenarios and interviews before reviewing the manuscript.

#### Sample Size

Finally, the sample size was relatively small with 7 participants, who were heterogeneous in terms of roles. The number of participants needed for usability studies is controversial. While some authors consider 5 participants sufficient due to the descriptive nature of these studies [41,42], more recent approaches advocate for larger sample sizes [43]. A reason for pursuing larger sample sizes is the nonhomogeneous probability of identifying specific use errors. Using the good-turning model modified by Hertzum and Jacobsen as outlined by Borsci et al [44], the adjusted estimated percentage of use errors discovered by an individual participant in our study was 23.8%. Following this, 85% of use errors are expected to be discovered with this study of 7 participants.

However, this estimation does not reflect saturation within the interview data. While the suitability of data saturation is controversial in the context of Braun and Clarke’s [45] thematic analysis [46], it should be acknowledged that this study’s design did not allow for ongoing assessment of saturation to guide further inclusion. Nevertheless, no unique codes were applied exclusively to the final interview transcript, suggesting that the contribution of additional participants for the ongoing analysis was diminishing. More importantly, although including additional participants will always have the potential to reveal new insights or deepen existing ones, the study’s findings provide actionable information to guide the further development of the EnzySystem, indicating data sufficiency.

### Overall Conclusion and Future Directions

The current findings demonstrate high acceptance across HCPs for the EnzySystem HemA, despite some critical limitations of the user interface. To evaluate the effectiveness of potential modifications and ensure the platform’s suitability for broader implementation, future usability evaluations should be conducted in realistic use environments and include participants from diverse care facilities. The universal demand among participants for faster results and improved testing to monitor treatment in people with hemophilia A underscores the urgency of these next steps, along with the need to assess the platform’s usability for people with hemophilia A.

## Supplementary material

10.2196/77621 Multimedia Appendix 1 Graphical user interface wireframe.

10.2196/77621 Multimedia Appendix 2 Quick reference instructions.

10.2196/77621 Multimedia Appendix 3 Interview guide.

10.2196/77621 Multimedia Appendix 4 Codebook.

10.2196/77621 Checklist 1 COREQ checklist.

## Abbreviations

COREQ: Consolidated Criteria for Reporting Qualitative Studies
EMR: electronic medical record
FVIII: factor VIII
HCP: health care provider
POCT: point-of-care testing
QRI: quick reference instructions
TGA: thrombin generation assay
