# Supplementary figures and images for: Measuring FVIII Activity and Thrombin Generation Simultaneously With a Novel Point of Care Platform (EnzySystem HemA): Qualitative Usability Evaluation
Source: JMIR Form Res. 2025 Oct 16;9:e77621. doi: 10.2196/77621 (PMC12530449; doi:10.2196/77621)

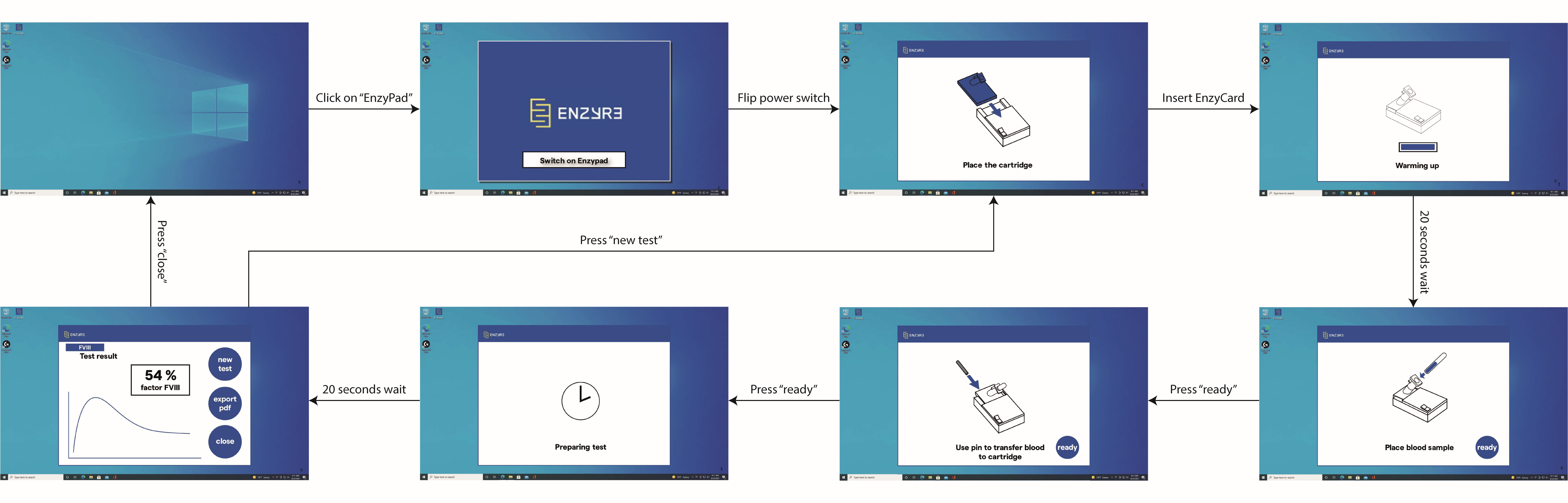

Supplement: Multimedia Appendix 1 [file formative-v9-e77621-s001.png]
