# Supplementary material for: Measuring FVIII Activity and Thrombin Generation Simultaneously With a Novel Point of Care Platform (EnzySystem HemA): Qualitative Usability Evaluation
Source: JMIR Form Res. 2025 Oct 16;9:e77621. doi: 10.2196/77621 (PMC12530449; doi:10.2196/77621)
Supplement: Multimedia Appendix 2 [file formative-v9-e77621-s002.pdf]

Quick Reference Instructions for EnzySystem HemA - Healthcare providers

RUO For Research Use Only.

Important Information

The EnzySystem HemA Version B is an *in vitro* diagnostic device used for the quantitative determination of Factor VIII activity. This test is intended for use only with specimens of 3.2% citrated whole blood obtained by venipuncture.

Healthcare providers must follow these instructions to perform the test

Read the entire Quick Reference Instructions before performing the test.

Warnings

The blood tube insert contains needles. Do NOT stick a finger in the blood tube insert, to prevent injury.
Do NOT use excessive force when inserting or removing the blood tube or the EnzyCard from the system
Wear the appropriate protective attire for your safety when handling patient samples.

Storage Specifications

Patient Samples
Do NOT use a stored patient specimen for this test

EnzyCards
(Only use when unused and in original packaging)

TEMPERATURE
2° - 8°C
35 - 47°F

Operating Conditions

TEMPERATURE	HUMIDITY	PRESSURE
**15° - 30°C**<br>59 - 86°F	**Max. 80%**	**70kPa – 106 kPa**

- Perform the test on a flat horizontal surface
- Perform the test immediately after specimen collection

Materials Provided

EnzyPad (reusable) + **EnzyCard** (disposable) =

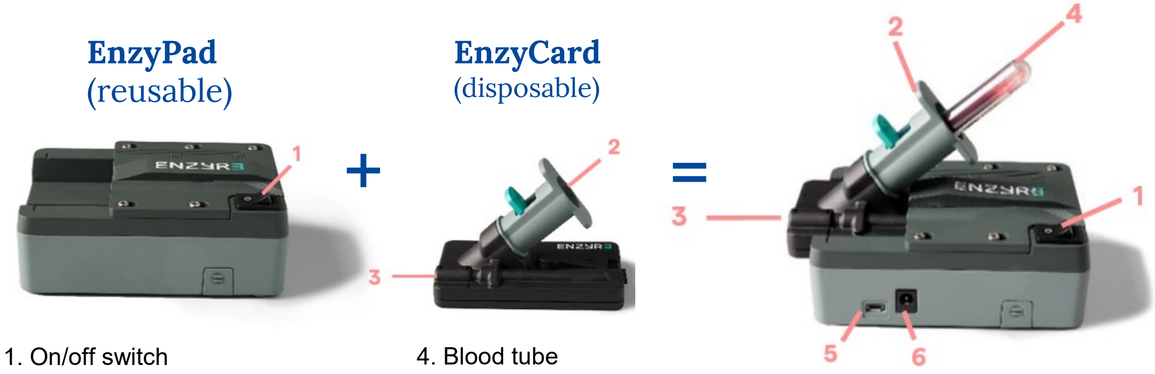

1. On/off switch
2. Blood tube insert
3. Syringe
4. Blood tube
5. USB micro port
6. Jack connector male port

EnzyApp

Application that collects data from EnzyFirmware, calculates the result and displays the data graphically on the screen

Materials not Provided

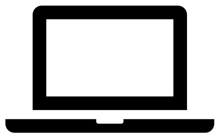

Tablet / stand-alone laptop/desktop

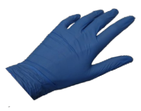

Gloves

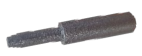

Plastic plug to press the syringe into the EnzyCard

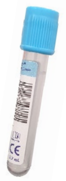

3.2% sodium citrate BD Vacutainer tubes (ref. 363083)

Preparatory handling

1. Turn on the laptop

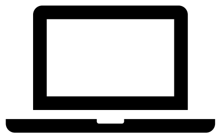

2. Start the application by clicking on the ''Enzypad'' icon on the screen.

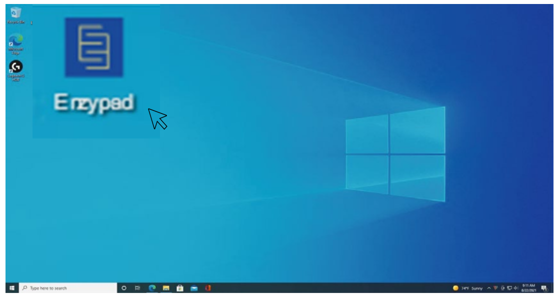

3. Turn ON the EnzyPad using the power switch on the top of the device.

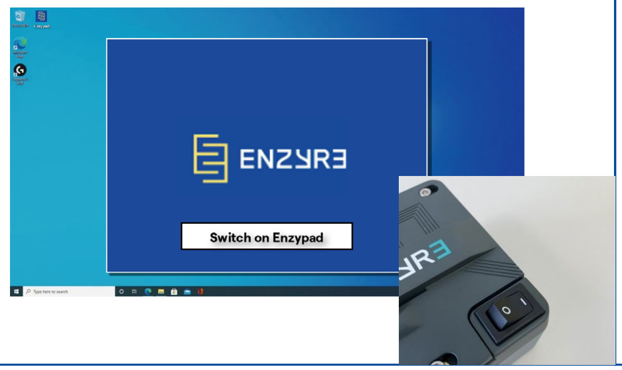

4. Collect blood using-a sodium citrated blood tube (Vacutainer, BD). This step must be performed by a trained person.

Note: Ensure proper mixing of the specimen by inverting the tube 10 times.

Perform a patient specimen test

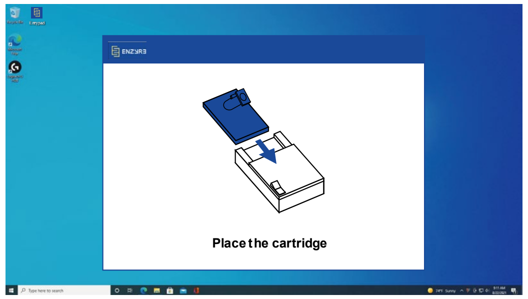

1. Slide the EnzyCard into the EnzyPad until the alignment lines of the two components are in the position as shown the figure below.

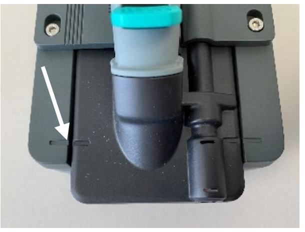

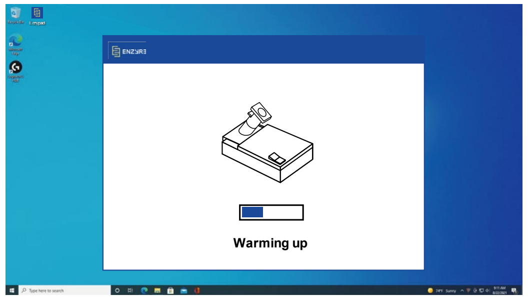

2. The software displays a message: Warming up. From this point, wait between 3 - 3.5 minutes until the device is ready.

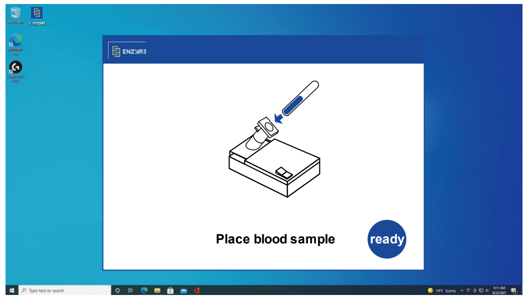

3. Hold the EnzyPad and EnzyCard in place with one hand. With the other hand, insert the blood tube. The blood tube will remain in this position for the remainder of the test

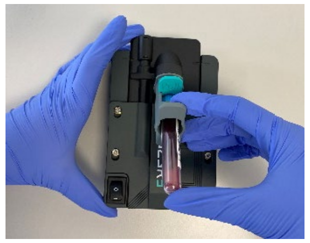

Press the “ready” button that appears on the screen.

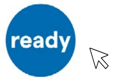

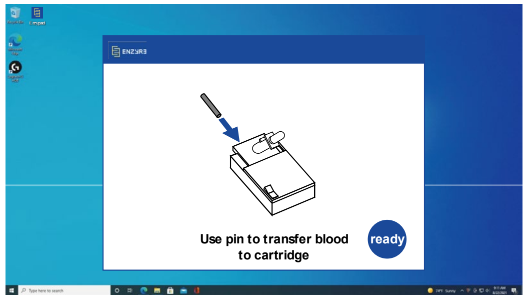

4. Use the plastic plug to press the syringe integrated in the EnzyCard: press the tip of the plug into the hole of the EnzyCard as shown below. The white marker goes from the first hole to the second.

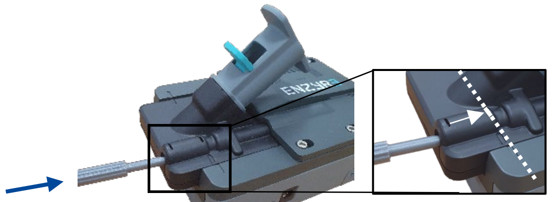

Press the “ready” button that appears the screen.

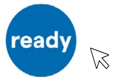

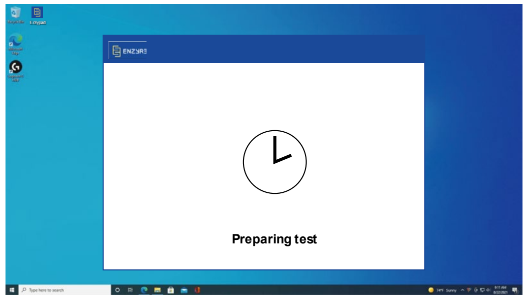

5. The measurement takes 60 minutes from the time the EnzyCard is inserted.

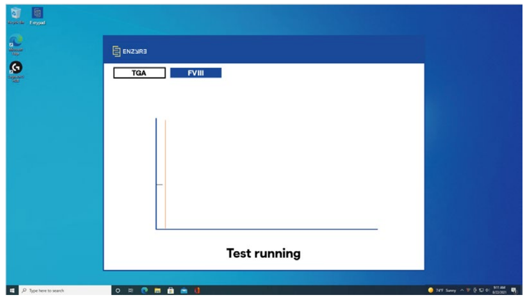

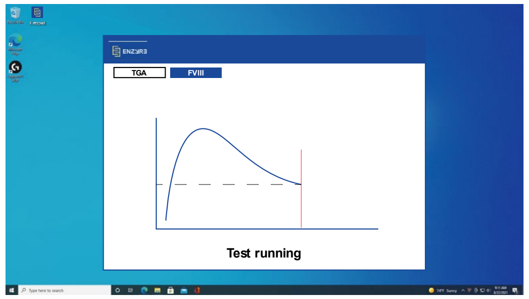

6. The result is displayed on the screen.

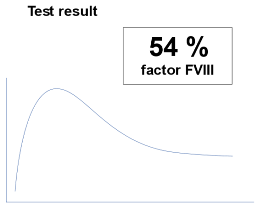

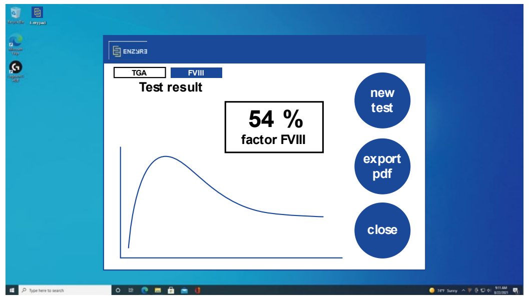

7. When the test is finished, you can choose to start a new test, export the test result as a PDF or close the program by clicking the appropriate button.

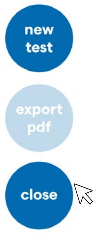

ENZYRE

Power OFF and Disconnect

**1**. Before disconnecting any part of the EnzySystem, ensure that a proper clinical waste container is nearby. 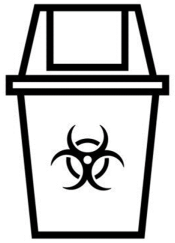	**2**. When finished measuring, turn off the EnzyPad by pressing the ON/OFF switch. 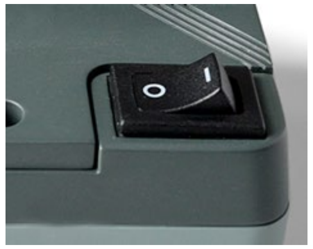	**3**. Remove the EnzyCard (including the blood tube) by holding the EnzyPad with one hand and sliding the EnzyCard out of the EnzyPad with the other hand.<br>Dispose the EnzyCard in the appropriate waste container. Avoid walking with a used EnzyCard. 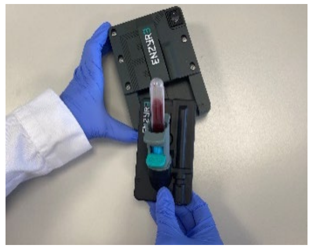

Possible Results

Result	Interpretation
% FVIII	The test was successful. The result is expressed as percent activity.
NO RESULT – REPEAT TEST	If the result is **NO RESULT- REPEAT TEST**, collect new specimen and **REPEAT TEST** with a new EnzyCard.
INSTRUMENT ERROR	If the result is **INSTRUMENT ERROR**. Press **CLEAR ERROR** and follow the on-screen instructions. When the Home screen appears, start a new test with a new specimen and new EnzyCard.

NOTE: If the external control gives an incorrect result, repeat the external control run. If repeated control runs do not produce the expected results, contact Enzyre Technical Support.

Enzyre BV
Transistorweg 5
6534-AT Nijmegen
The Netherlands
Tel: +31 (0)24 6690036
