# Supplementary material for: Measuring FVIII Activity and Thrombin Generation Simultaneously With a Novel Point of Care Platform (EnzySystem HemA): Qualitative Usability Evaluation
Source: JMIR Form Res. 2025 Oct 16;9:e77621. doi: 10.2196/77621 (PMC12530449; doi:10.2196/77621)
Supplement: Multimedia Appendix 3 [file formative-v9-e77621-s003.pdf]

Interview (30 min)

<Ask the following questions. Use follow up questions and ask for specific examples to illustrate the statement of the user.>
All questions are intended as open questions, checkmarks are used to make data processing easier.

INTERVIEW

C	Questions	
	**First impression**	
**Q-1.**	How did the test go?	
**Q-2.**	What is your impression of the device?	
**Q-3.**	Did you experience any difficulties?<br><br>Was the use of the device according to your expectations?<br><br>Were there any surprises	❑ Yes<br>❑ No<br><br>❑ Yes<br>❑ No<br><br>❑ Yes<br>❑ No
**Q-4.**	How would you evaluate the use of the product?	

C	Questions	
	**Look back at walk through**	
**Q-5.**	Shall we go through the steps again to evaluate with you what you encountered?<br><br>*< Conversation with participant, ask participant to explain experiences. Address what we saw.>*<br><br>*<Eg: “At the beginning*<br>- *you did this …*<br>- *did you know how to start ?*<br><br>- *In which step you experience any difficulties?*<br>- *Could you describe the situation?*<br>- *What do you think the cause was?”*	Steps which were experienced as difficult:

To assistant: do you want to add / ask something else?
To participant: can we continue to the detailed questions?

C	Questions	
	**Detailed questions on application**	
**Q-6.**	Do you have any concerns regarding the device, assuming that the measurement itself is correct?<br>- Which concern?<br>- In what situation this would lead to risk?<br>- What would be the cause?<br>- What would be the consequence? And what actions would you take if this happens?<br>- What could be improved to prevent this?	Concerns:

C	Questions	
**Q-7.**	Specifically, do you see any risks on blood contact?<br>If so:<br>- In what situation this would lead to risk?<br>- What would be the cause?<br>- What would be the consequence? And what actions you would take if this happens?<br>- What could be improved to prevent this?	❑ Yes<br>❑ No
**Q-8.**	How would you want to transfer the results to the EMR?	
**Q-9.**	We are curious if and how the device could fit in clinical practice.<br>In what situations do you currently measure FVIII levels?	Use cases:
**Q-10.**	For which of these situations do you think you could use the EnzySystem?	Use case:

C	Questions	
**Q-11.**	Do you think the EnzySystem as is would be suitable for this situation?<br>- Which activities would change in your workflow when you have the EnzySystem?<br>- What do you think is missing from the product as presented today to be able to do the measurement in this situation?<br>- What improvements would be needed?	❑ Yes<br>❑ No, changes needed
**Q-12.**	Currently the test takes up to 60 minutes. Would that be acceptable?<br>- If not, would 30 minutes be acceptable?<br>- If not, what would be acceptable?	❑ Yes, 60 min. is acceptable<br>❑ No, 60 min. is not acceptable<br>❑ ..... min. is acceptable
**Q-13.**	Do you think the product could be used in other situations?<br>(open question, not go through the options)	Mentioned by participant:<br>❑ Emergency care at hospital ER<br>❑ In hospital testing by nurses<br>❑ During surgeries<br>❑ Home use by patient<br>❑ Testing at general practitioner office<br>❑ Near patient testing in satellite hub<br>❑ Critical care – testing at trauma location<br>❑ Other:
**Q-14.**	What would be needed for implementation of the product in these situations?	❑ Nothing<br>❑ Specific consequences:

C	Questions	
**Q-15.**	Do you think training would be needed?	❑ Yes<br>❑ No
**Q-16.**	You mentioned a number of improvements,<br>(…, …, …)<br><br>Suppose all your input is implemented and we have an improved device:<br><br>- Can you summarize, what do you see as the benefits of this system?<br>- Would there still be down sides?	
**Q-17.**	Are there aspects we have not discussed and you would like to address?	
**Q-18.**	Do you have any questions for us?	

Thank you again, your input is very valuable!
[Name of research nurse] will guide you on your way back

Clean up (5 min)
< stop camera recordings >
< stop screen capture recording >
< swap SD cards>
<Reorganize test materials for next test.>
